# Pharmacological approaches to pulmonary fibrosis following COVID-19

**DOI:** 10.3389/fphar.2023.1143158

**Published:** 2023-06-15

**Authors:** Stefan Lassan, Tomas Tesar, Jana Tisonova, Monika Lassanova

**Affiliations:** ^1^ Department of Pneumology, Phthisiology and Functional Diagnostics, Slovak Medical University and Bratislava University Hospital, Bratislava, Slovakia; ^2^ Department of Organisation and Management of Pharmacy, Faculty of Pharmacy, Comenius University, Bratislava, Slovakia; ^3^ Institute of Pharmacology and Clinical Pharmacology, Faculty of Medicine, Comenius University, Bratislava, Slovakia

**Keywords:** pulmonary sequelae associated with COVID-19, post-COVID-19 pulmonary fibrosis, corticosteroids, antifibrotic agents, pharmacological treatment

## Abstract

**Background:** In the past few years, COVID-19 became the leading cause of morbidity and mortality worldwide. Although the World Health Organization has declared an end to COVID-19 as a public health emergency, it can be expected, that the emerging new cases at the top of previous ones will result in an increasing number of patients with post-COVID-19 sequelae. Despite the fact that the majority of patients recover, severe acute lung tissue injury can in susceptible individuals progress to interstitial pulmonary involvement. Our goal is to provide an overview of various aspects associated with the Post-COVID-19 pulmonary fibrosis with a focus on its potential pharmacological treatment options.

**Areas covered:** We discuss epidemiology, underlying pathobiological mechanisms, and possible risk and predictive factors that were found to be associated with the development of fibrotic lung tissue remodelling. Several pharmacotherapeutic approaches are currently being applied and include anti-fibrotic drugs, prolonged use or pulses of systemic corticosteroids and non-steroidal anti-inflammatory and immunosuppressive drugs. In addition, several repurposed or novel compounds are being investigated. Fortunately, clinical trials focused on pharmacological treatment regimens for post-COVID-19 pulmonary fibrosis have been either designed, completed or are already in progress. However, the results are contrasting so far. High quality randomised clinical trials are urgently needed with respect to the heterogeneity of disease behaviour, patient characteristics and treatable traits.

**Conclusion:** The Post-COVID-19 pulmonary fibrosis contributes to the burden of chronic respiratory consequences among survivors. Currently available pharmacotherapeutic approaches mostly comprise repurposed drugs with a proven efficacy and safety profile, namely, corticosteroids, immunosuppressants and antifibrotics. The role of nintedanib and pirfenidone is promising in this area. However, we still need to verify conditions under which the potential to prevent, slow or stop progression of lung damage will be fulfilled.

## Introduction

Since its appearance, Coronavirus-2019 (COVID-19) disease potentially resulted in over 766 million confirmed cases and over 6.9 million deaths globally by the end of May 2023 (World Health Organization, 2023c). We must take into account, that experience from autopsy cohorts indicates a higher number of deaths due to COVID-19 than officially reported (Schwab et al. 2022). Despite the undeniable progress, mortality due to COVID-19 is still unacceptably high, with respiration-related harms representing the second most common cause of death in hospitalized COVID-19 patients, accounting for 15.4% of cases (Elezkurtaj et al. 2021; Centers for Disease Control and Prevention, 2022). Certain approaches to post-mortem diagnosis can refine the causes of death, expand the understanding from clinical point of view and contribute to the proposal and implementation of targeted measures (Pomara et al., 2020b).

The COVID-19 pandemic may be considered a major threat to public health, with devastating social and economic impacts. The last 2 years have brought tremendous progress in the field of COVID-19 prevention, treatment and far-reaching public health interventions (Zhou et al., 2021; Ayouni et al., 2021). Thanks to global efforts, The World Health Organization (WHO) announced on 5 May2,023 that it was ending the global health emergency declared for COVID-19 more than 3 years ago. The WHO emphasized, that the decision does not signal the end of the pandemic and there is no reason to cancel COVID response systems (World Health Organization, 2023b). On the contrary, stakeholders from the field of healthcare and politics are obliged to pay attention to the “COVID-19 survivor” management with several issues which arose as a result of (multi)organ damage and require the best possible comprehensive medical care in order to improve survival and quality of life (Pomara et al., 2020a; Sessa et al. 2020).

Pharmacological approaches to prevention and treatment played an indisputable role during the fight against the burden of the pandemic. Completed and ongoing trials have been proposed for verification of tailored pharmacological strategies covering virtually every aspect of the disease (Heustess et al. 2021). As a result, the amount of evidence is growing for various drugs, biologics, vaccines, convalescent plasma therapy, monoclonal antibodies, immunoglobulin therapy, cell therapy, etc. (Panahi et al., 2023). As new treatments towards the reduction of early disease mortality have emerged, the long-term consequences of severe COVID-19 have grown in importance (O´Mahoney et al. 2023). In this regard, reports of worrying long-term respiratory complications have been increasing since the beginning of the pandemic (Schlemmer et al. 2023). Clinical practice recommendations directed at the mid- and long-term consequences of the initial disease continue to pose an important task for the future (Greenhalgh et al. 2020). This literature review aims to discover the main mechanisms involved in the pathogenesis of the COVID-19-associated respiratory sequelae with emphasis on interstitial lung disease. We summarized the substantial knowledge regarding the treatment strategies based on conventional, repurposed and innovative drugs, with focus on corticosteroids and antifibrotic agents.

## Methods

This literature review was conducted through World Health Organization (WHO), Google Scholar, PubMed/MEDLINE, Web of Science, and EMBASE databases to identify pertinent publications: studies, scientific papers and other relevant sources published up to 25 May 2023. Additional relevant articles were identified from the review of citations referenced. Due to the lack of randomized controlled trials, we have also included letters to the editor, case reports, case series, and review articles. The search term has been limited to the English language and the availability of the full text and abstracts. Sources with unsatisfying relevance to the topic of the review or not adequately reporting the objectives and conclusions were excluded. The search was done using these keywords: (SARS-CoV-2 OR COVID-19 OR Coronavirus 2019) AND (Post-COVID-19 pulmonary sequelae) AND (fibrotic OR fibrosis OR interstitial lung disease) AND (pharmacological treatment OR pharmacotherapy).

### Post-COVID-19 pulmonary fibrosis

Real-life clinical experience has shown us that the clinical outcome of SARS-CoV-2 infection varies on the intra- and interindividual level. The diversity of clinical symptoms and manifestations originating from the multiple organ systems involvement, underlying pathophysiology and comprehensive management during the acute phase of COVID-19 are well described elsewhere (Wiersinga et al., 2020; NIH, 2023). One of the most significant unmet needs lies in the treatment of pulmonary consequences after SARS-CoV-2 infection, ranging from non-serious and spontaneously resolving abnormalities to symptomatic persistent lung illness. A recent systematic review of 57 studies comprising more than 250,000 survivors of COVID-19 showed that the most prevalent post-acute sequelae comprised respiratory disorders with chest imaging abnormalities, pulmonary diffusion abnormalities, increased oxygen requirement and persistent dyspnoea or cough (Groff et al., 2021). Previous studies conducted on a number of viral respiratory infections (e.g., Influenza, SARS, MERS) have confirmed a close relationship between lung tissue injury, subsequent abnormal inflammatory response and the appearance of certain lesions with a tendency of progression to fibrosis (Udwadia et al. 2021). Radiological abnormalities, in hand with pulmonary functional involvement and altered health status, persisted as long as 2 years after the onset of SARS or MERS infection (Ngai et al., 2010; Das et al., 2017). Moreover, in 4% of patients, severe viral pneumonia due to SARS infection resulted in the development of pulmonary fibrosis, the clinical and radiological signs of which were still evident even after 15 years of follow-up (Zhang et al. 2020). With reference to a meta-analysis of 46 studies evaluating radiological changes and 50 studies focusing on lung function impairment, distinct sequelae consistent with parenchymal lung disease following viral pneumonia were common (e.g., SARS-CoV-2, SARS, MERS, Influenza, etc.). In a subgroup infected with SARS-CoV-2, after a median follow-up of 3 months, inflammatory lesions were still visible in 50%, fibrotic signs in 29% and impaired gas transfer according to lung function tests in 38% of patients, respectively (Fabbri et al., 2022). Nevertheless, most patients who have survived severe COVID-19 gradually improve over time. Reports from several observations indicate a trend towards improvement of dyspnoea, performance status and exercise capacity. However, even after 6–12 months, there is still a non-negligible group of patients showing evidence of persistent physiological and radiographic change (Wu et al., 2021; Sonnweber et al., 2022).

Although definitive fibrosis is not evident in most fatal COVID-19 cases, multiple findings (e.g., consolidations) on computed tomography (CT) images obtained before death are known to be associated with more severe disease developing over time (Ye et al. 2020). *Barisione et al.* described the chronology of lung pathological changes in severe fatal COVID-19. The authors collected lung tissue samples using a transbronchial lung cryobiopsy carried out in ventilated lungs immediately after death. The histopathological findings were subsequently correlated with CT patterns obtained earlier. The early/exudative phase was characterised by the occurrence of ground-glass opacities and mid/proliferative lesions with crazy-paving patterns. On the other hand, the late/fibrous phase was associated with the consolidation pattern usually localised in the lower/middle lobes (Barisione et al., 2021).

With regard to diffuse alveolar damage and often aggressive treatment of respiratory failure (e.g., ventilator-induced lung injury), the risk of major lung injury with potential aberrant healing leading to fibrosis must be kept in mind, particularly in severe COVID-19 patients (Li et al., 2021). Initial data from the early phase of the pandemic suggested that up to 17% of COVID-19 patients may have suffered from some degree of pulmonary fibrosis emerging from severe pulmonary damage (Pan et al. 2020). Following COVID-19 pneumonia, the estimated percentage of survivors with permanent fibrotic-like changes or parenchymal lung abnormalities varies between studies: from 72% in those who could be liberated from mechanical ventilation (McGroder et al. 2021), through 35% in those with a severe acute phase (Han et al. 2021), to 4.8% among all patients discharged from hospital (Myall et al. 2021). Moreover, based on the historical observational data on SARS patients, 2%–6% of patients who have recovered from moderate to severe COVID-19 may be threatened by fibrotic remodelling of lung tissue. *Stewart et al.* newly published results from the UKILD Post-Covid study with more than 3,700 patients who were discharged from hospital after confirmed COVID-19. The presence of residual lung abnormalities on CT scans during the follow-up period (median 113 days) was reported in 11% of encompassed participants (Stewart et al. 2023). In this context, *Bazdyrev et al.* estimated the prevalence of COVID-associated fibrosis between 10 and 15 patients per 10,000 people in the general population, which would represent tens of times higher risk compared to idiopathic lung fibrosis (Bazdyrev et al. 2021). As findings coming from imaging techniques reflect pathophysiological lung impairment, patients with radiologically confirmed lesions in the course of the acute phase of the disease in particular are at risk (Table 1) (Tobin. 2020; Barbeta et al., 2020). Similar observations apply to the relationship between functional status of the respiratory system and morphological changes. Therefore, identifying abnormalities in pulmonary function tests (PFTs) during the follow-up period after recovering from COVID-19 can predict the risk of pulmonary fibrosis (Table 2) (Gentile et al. 2020). Given the heterogeneity of the survivors with COVID-19 pneumonia, the respiratory community calls for a systematic pulmonary assessment including PFTs (Raghu and Wilson. 2020). Pulmonary function testing is a useful procedure in detecting the functional impairment caused by interstitial lung involvement. Using serial PFTs in order to assess the individual behaviour is a widely recommended strategy derived from the management of ILDs irrespective of their primary aetiology. The rate of decline in lung function and exercise capacity reflects the course of lung fibrosis along with the response to therapy and is considered a key feature of progressive fibrosing phenotype (Kolb and Vasakova. 2019).

**TABLE 1:** Radiological findings on lung computed tomography in patients with severe SARS-CoV-2 infection during the acute phase and post-acute period (Marvisi et al. 2020; Guler et al. 2021; Tanni et al. 2021; Amin et al., 2022; Melhorn et al. 2022).

Acute phase	Post-acute and chronic phase
Ground-glass opacities	Architectural distortion
Reticular opacities	Parenchymal band
Consolidations	Traction bronchiectasis
Crazy-paving pattern	Reticular opacities or septal bulging and thickening
Organising pneumonia pattern	Ground-glass opacities
Diffuse alveolar damage pattern	Mosaic attenuation
Nodular opacities	Traction bronchiectasis
Halo sign, Reverse halo sign	Honeycombing
Pneumothorax/pneumomediastinum, pneumatocele	Reduction in lung volume
Lymph node enlargement	Pleural adhesions
Cavitation	
Pleural effusion	

**TABLE 2:** Functional findings associated with post-COVID-19 respiratory sequelae (adapted; based on Tanni et al. 2021).

Lung function	Exercise
DLCO ↓↓	VO2 ↓
KCO ↓	6MWD ↓
TLC ↓	Exercise capacity ↓↓
FEV1 ↔ ↓	Physical activity ↓
FVC ↓↔	

DLCO, diffusion capacity for carbon monoxide; KCO, transfer coefficient; FVC: forced vital capacity; FEV1, forced expiratory volume in the first second; TLC, total lung capacity; VO2, peak oxygen uptake; 6MWD, 6-min walk distance.

There is currently no common, generally accepted definition of pulmonary fibrosis arising from pulmonary involvement caused by severe SARS-CoV-2 infection. With reference to Tanni et al., Post-COVID-19 pulmonary fibrosis can be defined as the presence of persistent fibrotic changes associated with functional impairment, such as architectural distortion, parenchymal bands, ground-glass and reticular opacities, traction bronchiectasis and honeycombing, identified on follow-up CT scans (Tanni et al., 2021; Huang et al., 2021).

Despite growing evidence, many questions addressing the pathogenesis of Post-COVID-19 pulmonary fibrosis still remain unanswered (Bergantini et al. 2022). Autopsies can be helpful to improve this condition: They play, as a diagnostic tool, a pivotal role not only in determining the cause of death, but represent a procedure for revealing the mechanisms of virus-induced organ damage (Pomara et al., 2020a). Acute COVID-19 can lead to a wide range of clinical manifestations, including those with potentially fatal consequences—acute respiratory distress syndrome (ARDS), macrophage activation syndrome (MAS), haemophagocytic lymphohistiocytosis, or “cytokine storm” (Vardhana and Wolchok. 2020). Alveolar epithelial injury is considered the decisive primary insult. (Nalk and Moore. 2010). The local proinflammatory environment of macrophage and immune cell infiltration in the lung can outbalance the natural homeostatic tissue repair functions, resulting in irreversible damage (Zhang et al. 2021). In the course of severe inflammation, counterregulatory mechanisms are synchronously engaged in order to avoid excessive tissue destruction. The involvement of these mechanisms is associated with the rise in local and systemic levels of suppressive cytokines, such as IL-10 and Transforming growth factor β (TGF-β). In consequence, inappropriately accumulated fibroblasts and myofibroblasts from multiple sources start to produce and deposit connective tissue components, which leads to further morphological and functional disruption (Hall et al., 2022). Concurrently, common fibrosis-related pathways (mainly the TGF- β signal pathway, the WNT signal pathway and the YAP/TAZ signal pathway) play a supporting role (Piersma et al. 2015). Usually, this pro-fibrosis sequence will be turned off when the insult stops. If imbalanced or prolonged, this process results in aberrant wound healing and possibly self-perpetuating fibrotic response (Kumar et al. 2021). During this shaky phase of the disease, changing patterns on CT scans appear and potentially predict early evolution of pulmonary fibrosis (Ye et al., 2020; Tsujikawa et al., 2021).

Briefly summarised, multiple mechanisms are involved and play role in the remodelling of pulmonary tissue: ageing-related mechanisms (altered cellular communication, stem cell exhaustion), the renin-angiotensin system—with downregulation of ACE2 after virus binding, resulting in increased pro-inflammatory and pro-fibrotic activities, epithelial and endothelial to mesenchymal transition, a wide spectrum of upregulated effector molecules (interleukins—IL-1, IL-6, IL-8, CXCL10, MCP-3, CXCL13, matrix metalloproteinases—MMP 1,7, etc.), neutrophil extracellular traps, Galectin-3 mediated pathways, reactive oxygen species, induction of proliferation/migration and fibroblast activation and increased production of extracellular matrix components driven by fibrogenic mediators (TGF-β, vascular endothelial growth factor, insulin-like growth factor, osteopontin, periostin, etc.) (Wigén et al., 2020; McDonald, 2021; Tanni et al., 2021; Mohammadi et al., 2022; Bergantini et al., 2022). A high rate of systemic vascular dysfunction, especially pulmonary vasculopathy and the resultant hypoxic remodelling, may also be involved (Ackermann et al., 2020; Menter et al., 2020; Myall et al., 2022).

The identification of potential risk factors for the development of pulmonary fibrosis associated with COVID-19 remains a key task, although such risk factors have not yet been completely defined. A number of recently published studies intended to find potential risk factors and biomarkers in relation to this issue. Some of them are associated either with the individual patient profile (e.g., male gender, older age, active smoking, history of chronic respiratory or cardiovascular disease) or are related with the course/severity of SARS-CoV-2 infection: respiratory failure with need for high-flow-oxygen or invasive mechanical ventilation with high plateau pressures, exposure to high concentrations of supplemental oxygen, severity of acute infection, longer hospitalisation period, respiratory distress syndrome, dysregulated and protracted systemic inflammation, steroid/antibiotic/immunoglobulin treatment, fever, respiratory symptoms (especially shortness of breath), lymphopenia, neutrophile-to-lymphocyte ratio, high level of lactate dehydrogenase, elevated biomarkers of alveolar epithelial cell damage and fibroproliferation, and others (Tanni et al., 2021; Huang et al., 2021; Arnold et al., 2021; Sonnweber et al., 2022; Amin et al., 2022; Mohammadi et al., 2022). Genome-wide association studies have identified multiple genes involved in the development of ILDs (Allen et al., 2020). Such pre-disponing genetic alterations can drive the progressive course of pulmonary fibrosis triggered by SARS-CoV-2 infection (John et al. 2021).

Nevertheless, there is still a gap of knowledge concerning the natural course of Post-COVID-19 pulmonary fibrosis. The main clinical question is whether signs of early fibrosis will worsen or regress over time either spontaneously or with treatment. Furthermore, the impact of individual susceptibility should be taken into account (Wigén et al. 2020). At the same time, there is an active discussion about the need or timing for drug treatment of fibrotic lung abnormalities in COVID-19 survivors in the light of the possibility of spontaneous resolution. The European Respiratory Society statement on long COVID-19 emphasises the importance of follow-up strategies related to both pulmonary physiology and imaging in assessing long-term lung sequelae (Antoniou et al. 2022). Such targeted strategies could help identify individual disease behaviour, progression patterns and risk factors predictive for the evolution of Post-COVID-19 pulmonary fibrosis. Based on the experience from other interstitial lung diseases (ILDs), the trajectory of the disease can be very heterogeneous, with patients having both progressive and stable periods and even improvement according to follow-up PFTs (Hoffmann-Vold et al. 2021).

It has to be stated that previously published research reports have usually considered post-COVID lung disease to be a single entity (e.g., Post-COVID-19 pulmonary fibrosis). There is still a lack of pertinent findings supported by histopathological examinations of tissue specimens obtained from living or deceased subjects. Performing autopsies provides benefits in this direction and should be encouraged (*Pomara et al. 2020-2*). In a recent prospective analysis of morpho-phenotypical changes, *Ravaglia et al.* identified three different subtypes obviously based on different COVID-19-induced pathways: lung fibrotic appearances and signs indicating the occurrence of interstitial lung disease (ILD) before SARS-CoV-2 infection, an auto-inflammatory phenotype suggesting an ongoing (sub)acute stage of the disease and, finally, patients with minimal changes on lung CT scans with histologically confirmed diffuse vascular abnormalities (Ravaglia et al. 2022). In addition, Michalski et al. newly described a specific pattern characterised by a predominant small airway impairment (Michalski et al. 2021). The finding of air-trapping on serial inspiratory/expiratory thin-section CT scans supports the suspicion of Post-COVID-19 small airway disease (Franquet et al. 2022). The corresponding level of small airway dysfunction can be confirmed during PFTs or with help of other methods for assessing the most distal airways (Cherrez-Ojeda et al. 2022). In summary, the spectrum of pulmonary sequelae associated with COVID-19 further remains an object of interest, owing to the population-wide impact of these variable manifestations, which may require an individual approach.

Finally, based on previously mentioned evidence, we hypothesize the origination of Post-COVID-19 pulmonary fibrosis as follows:

1. The rapid development of pulmonary fibrosis as a result of unique direct coronavirus-induced intense lung injury and subsequent dysregulation of the immune response (e.g., cytokine storm). As an analogous example serves acute respiratory distress syndrome/diffuse alveolar damage/acute interstitial pneumonia originating from a variety of infective or non-infective causes (Batah and Fabro. 2020; Cardinal-Fernández et al., 2017). Nevertheless, progressive pulmonary fibrosis is not a typical feature of ARDS and the overall prognosis of survivors is favourable (Masclans et al. 2011). In case of COVID-19 ARDS is the situation less clear (Chaudhary et al. 2020), but available data show, that lung fibrosis can persist in some patients across different time periods (van Gassel et al. 2021).
2. The involvement and predominance of aberrant healing processes without clear dependence on the intensity and frequency of the primary insult. Accelerated aging and senescence of the pulmonary tissue could play a role, a characteristic feature of which is the formation of functionally unequal fibrotic tissue. As an analogous example serves a bright group of interstitial lung diseases with progressive fibrotic phenotype (e.g., usual interstitial pneumonia, non-specific interstitial pneumonia or hypersensitive pneumonitis) (Raghu et al. 2022).
3. Unmasking of a latent ILD, already smouldering by the time the coronavirus infection broke out. Or, the coronavirus acts as a last piece in the puzzle of insults waking up the process in predisposed individuals (Ravaglia et al., 2022; Patrucco et al., 2023).

### Pharmacological approaches to prevent or treat post-COVID-19 pulmonary fibrosis

To our knowledge, there is currently no clear consensus regarding the treatment of a patient with interstitial pulmonary involvement due to COVID-19. We assume, that the treatment in daily clinical practice mostly relies on experience and expertise gained with other ILDs or derived from recommendations prepared for them. In contrast, several well prepared clinical guidelines intended for the acute phase of COVID-19 have already been published since the beginning of the pandemic (NIH, 2023; World Health Organization, 2023a; Roche et al., 2022). The extraordinary burden on healthcare systems led to an accelerated search for solutions directed at the entire sequence of disease phases—from initial acute disease to long-COVID clinical manifestations and pathophysiological disturbances. Numerous drug trials have been conducted with either new licenced treatments or repurposed drugs. However, priorities established for allocating scarce resources have raised a number of ethical questions (Emanuel et al. 2022). Nowadays, repurposed drugs continue to occupy a key position in the proposed algorithms used in the treatment of a wide spectrum of COVID-19 patients. The use of repurposed drugs under the conditions of a global pandemic inevitably brings different simultaneous effects. Principally, the fairness of resource distribution becomes an issue that must be taken into account. From an ethical point of view, the rights of original indication patients to receive treatment (often suffering from a lack of supplies due to high demand) must be secured. While certain repurposed drugs are no longer a topic of discussion, as clinical efficacy and safety failed to be confirmed (e.g., anti-HIV antivirals, chloroquine, colchicine, ivermectin, fluvoxamine, etc.), several others have found widespread clinical use. Essential antivirals (molnupiravir, remdesivir) or anti-inflammatory/immunomodulating drugs (corticosteroids, anti-IL-6 monoclonal antibodies, JAK/STAT inhibitors, etc.) belong to the latter group. Therefore, therapeutic strategies should be guided by conclusive evidence instead of loose hypothesis or empirical decisions (Ino et al. 2021).

Certain ILDs with an intrinsic tendency to fibrotic tissue accumulation can manifest in the form of a progressive phenotype (Kolb and Vasakova. 2019) and include idiopathic nonspecific interstitial pneumonia, connective tissue disease-associated ILDs (e.g., associated with rheumatoid arthritis, systemic sclerosis, polymyositis/dermatomyositis), hypersensitivity pneumonitis, unclassifiable idiopathic interstitial pneumonia, ILDs related to other occupational exposures and sarcoidosis (Raghu et al. 2022). Corticosteroids and/or immunosuppressants are still among the most commonly used drugs in the treatment of these diseases. However, such an approach is often empirical in nature and may not always meet efficacy and safety criteria (Richeldi et al. 2018). There is growing evidence regarding similarities between certain characteristics of severe COVID-19 and lung manifestations of autoimmune disease (connective tissue disease-associated ILDs). Overlapping areas of similarity include serological, clinical, radiological and even histopathological features. The presence of specific antinuclear antibodies (ANAs) predicts adverse clinical outcomes, e.g., respiratory failure due to ARDS, the need for intensive-care unit admission or invasive mechanical ventilation. Thus, autoimmune mechanisms are presumably involved beyond the acute phase of SARS-CoV-2 infection (Gagiannis et al., 2020).

With regard to the imminent pandemic of Post-COVID-19 pulmonary fibrosis, a wide spectrum of potential medications is under research whose overview is summarised in Table 3.

**TABLE 3:** Innovative and repurposed medications in research for pharmacological treatment of Post-COVID-19 pulmonary fibrosis (Echeverría-Esnal et al., 2021; Kumar et al. 2021; Bazdyrev et al. 2021; Cherrez-Ojeda et al. 2022; Mohammadi et al. 2022; Wu et al. 2020; Zheng et al. 2023).

Medication	Characteristics
Spironolactone	Spironolactone acts through spironolactone-dependent upregulation of the adenosine A2A receptor, which plays an important role in the epithelial-mesenchymal transition process. Spironolactone could modulate the extracellular matrix synthesis through its receptors
Azithromycin	The macrolide antibiotic with pleiotropic properties inhibits the proliferation of fibroblasts, collagen synthesis, TGF-β synthesis with the corresponding suppression of pro-fibrotic genes, the PI3K/AKT and JAK/STAT signal pathways which are involved in the fibrotic tissue remodelling
Colchicine	An anti-inflammatory drug that inhibits the synthesis of TNF-α, IL-6, monocyte migration and secretion of MMP-9. All of these effects undermine the fibrotic impact of severe COVID-19
Treamid	Treamid is a bis-amide derivative of dicarboxylic acid (BDDA). Treamid causes anti-inflammatory and antifibrotic effects by suppressing the production and deposition of collagen and stimulating endothelial progenitor cell synthesis
Deupirfenidone	N-aryl pirfenidone derivate with higher expected efficiency compared with pirfenidone. Inhibits various proinflammatory mediators, such as IL-6, TNF-α and TGF-β. Presumably with better gastrointestinal tolerance
Collagen-Polyvinylpyrrolidone	Drug with broad effectiveness for supressing multiple cytokines during cytokine storm in COVID-19; stimulates the suppression of tissue fibrosis via the TGFβ-pathway
Bovhyaluronidase Azoximer (Longidase)	Bovhyaluronidase azoximer is a long-acting hyaluronidase with antifibrotic properties due to the disruption of collagen fibre formation through the degradation of glycosaminoglycans by hyaluronidase
Genistein	Genistein is a soy isoflavone with a structure similar to that of oestrogen (phytoestrogen). Genistein suppresses the expression of transcription factors (NF-κB) and c-Myc and can contribute to cell cycle arrest by inducing the expression of cell cycle inhibitor proteins
Tetrandrine	Tetrandrine is a natural alkaloid, a major active component of the *Stephania tetrandra* plant. Tetrandrine shows multidirectional action, affecting reactive oxygen species, calcium channels and caspase-dependent pathways, with subsequent antitumour, anti-inflammatory and antiviral activity. Moreover, tetrandrine TET can lower type I and III collagen mRNA and collagen deposition in lungs
Anluohuaxian	Anluohuaxian is recommended for the treatment of liver fibrosis in China. Anluo-huaxian is known to suppress the expression of type I and III collagen mRNAs, the tissue inhibitor of matrix metalloprotease-1 (TIMP-1) and TGF-β1 in patients with liver fibrosis
Stromal Vascular Fraction (cSVF)	Regenerative medicine based on stromal vascular fraction cells (cSVF) derived from adipose tissue. cSVF is known to help mitigate damage from severe inflammatory disorders, provide immunomodulation and promote repair and regenerative effects
IN01 vaccine	Anti-EGF polyclonal neutralising antibodies, thereby preventing the binding of this factor to its receptors, blocking the signalling of pathways related to the growth of tumour growth factor and fibrosis
Chitotriosidase Inhibitor OATD-01	Chitotriosidase (chitinase 1) was shown to enhance TGF-β1-stimulated fibrotic responses
PRM-151	Recombinant form of the serum Amyloid P
Pamrevlumab	Monoclonal antibody against CTGF (connective tissue growth factor)
GLPG1690	Autotaxin and lysophosphatidic acid inhibitor
Resveratrol	Resveratrol, a natural polyphenol, is known to exert several pharmacological effects on lung injury. The antiviral efficacy of Resveratrol was demonstrated for several viruses, including coronavirus. Resveratrol was shown to mitigate the major pathways involved in the pathogenesis of SARS-CoV-2, including regulation of the renin-angiotensin system (RAS) and expression of angiotensin-converting enzyme 2 (ACE2), stimulation of immune system and downregulation of proinflammatory and profibrotic cytokines release
Human embryonic stem cell–derived immunity- and matrix-regulatory cells (hESC-IMRCs)	Stem cell therapy based mitigation of acute lung injury with subsequent pulmonary fibrosis
PLN-74809, TD139	Novel antifibrotics influencing different target molecules of the TGF-β pathway (ɑvβ6 integrin, galectins)
ɑvβ6 integrin inhibitor	Blockade of ɑvβ6 integrin-dependent latent TGF-β activation

### Corticosteroids

Despite the lack of conclusive efficacy data, systemic corticosteroids are often prescribed for ILDs other than idiopathic pulmonary fibrosis (IPF). Unclear evidence supports the recommendation for corticosteroid treatment in rheumatoid arthritis-ILD (RA-ILD) or idiopathic non-specific interstitial pneumonia, while in pulmonary sarcoidosis corticosteroids are the established choice (Maher and Wuyts. 2019).

Corticosteroids became the cornerstone pharmacological treatment for patients with severe lung disease in the course of SARS-CoV-2 infection. Within the RECOVERY study, a population of COVID-19 patients suffering from severe disease benefited from the use of low-dose dexamethasone during hospitalisation. The treatment resulted in a significantly lower 28-day mortality among those who were either mechanically ventilated or needed oxygen alone (The RECOVERY, 2021). Following convincing clinical experience, dexamethasone has been approved for the treatment of COVID-19 and adopted by generally accepted clinical guidelines based on conclusive evidence (NIH, 2023; Agrawal et al., 2020).

While most patients promptly recover from SARS-CoV-2 infection, for many the course of the disease becomes fulminant with adverse outcomes, potentially leading to death. The later stage of the disease is especially characterised by severe local and systemic immune responses bringing devastating effects to the host (Hall et al. 2022). There is an assumption that the mitigation of the acute phase inflammatory response and associated lung damage will lead to a lower probability of following consequences, mainly Post-COVID-19 pulmonary fibrosis.

Based on broad anti-inflammatory genomic and non-genomic actions, pulse corticosteroid therapy (>250 mg of prednisone equivalent per day for ≥1 day) is frequently administered in severe and/or life-threatening diseases arising from dysregulated immuno-inflammatory response to various insults (Sinha and Bagga, 2008; Buttgereit et al., 2004). The clinical course of severe COVID-19 pneumonia, as mentioned above, suggests the need for anti-inflammatory intervention beyond the standard low dose of dexamethasone. Salvarani et al. conducted a randomised double-blind, placebo-controlled trial with the aim of evaluating the efficacy and safety of pulse methylprednisolone therapy as an alternative to standard treatment. The 304 enrolled patients received 1 g of methylprednisolone intravenously for three consecutive days or a placebo in addition to standard dexamethasone. The pulse therapy failed to show benefits both in primary (duration of hospitalisation) or secondary outcomes (survival free from invasive ventilation, overall survival) (Salvarani et al. 2022). These results did not meet the expectations that had arisen from another study carried out in the early phase of the pandemic. Edalatifard et al. found out that a methylprednisolone pulse could represent an effective treatment for hospitalised patients with severe pulmonary involvement due to COVID-19. During a single-blind, randomised controlled trial, 34 patients from the active study group received methylprednisolone at comparatively lower doses—250 mg per day intravenously for consecutive 3 days. The proportion of patients with clinical improvement was higher in the methylprednisolone group than in the standard care group (94.1% versus 57.1%). Furthermore, the mortality rate was significantly lower in the methylprednisolone group (5.9% versus 42.9%; *p* < 0.001) (Edalatifard et al. 2020). Myall et al*.* made an important contribution to knowledge about the treatment of patients with persistent inflammatory interstitial lung disease following SARS-CoV-2 pneumonitis. The investigators conducted an observational study in a cohort of persistently symptomatic patients, where a significant proportion was identified as having Post-SARS-CoV-2 ILD with both radiological abnormalities and ongoing physiological and functional deficit. Six weeks after discharge from hospital, following a structured assessment, patients who met the eligibility criteria were treated at a maximum initial dose of 0.5 mg/kg prednisolone/day (the average starting dose was 26.6 mg/day) with a rapid dose reduction in the course of the following 3–6 weeks. Thirty patients completed the treatment with corticosteroids, which was well tolerated and associated with rapid and significant improvement in lung functions (PFTs), symptoms, markers of systemic inflammation and regression of abnormalities on chest CT (Myall et al. 2021). The single-centre, randomised, open-label COLDSTER trial recently enrolled adult subjects 3–8 weeks after acute COVID-19 symptom onset with persistent dyspnoea or resting hypoxaemia or exertional desaturation at screening and diffuse abnormalities involving ≥20% of lung parenchyma on CT. Study participants were divided into two study arms and received either high-dose prednisolone (40 mg/day for 1 week, followed by 30 mg/day for 1 week, 20 mg/day for 2 weeks and 10 mg/day for 2 weeks) or low-dose prednisolone (10 mg/day for entire 6 weeks). The authors concluded that high-dose prednisolone was not superior to the low-dose prednisolone in improving the clinical, radiological, physiological and patient-related outcomes (health-related quality of life, HRQL) (Dhooria et al. 2022).

The success of corticosteroid treatment, similarly to other COVID-19 medications, appears to depend on proper patient selection, timing, as well as the individualised dosing regimen. A high corticosteroid dose by itself does not guarantee treatment success, since medium- and long-term administration of low doses probably has the same effect. In addition, the role of systemic corticosteroids, mainly during the post-acute and long-COVID-19 period, needs further clarification. Currently, we can rely on rather heterogenous studies in terms of study design and patient inclusion criteria. Several placebo-controlled trials of corticosteroids are required to better inform clinical practice for treating at-risk patients or those with confirmed Post-COVID-19 pulmonary fibrosis. In this context, the role of other immunosuppressants remains uncertain in like manner. It can be assumed that at least patients with auto-inflammatory phenotypes would benefit from such treatment.

### Antifibrotic agents

The key characteristic of pulmonary fibrosis rests in an uncontrolled accumulation of extracellular matrix and cellular elements (e.g., fibroblasts, myofibroblasts) in the lung parenchyma. Pathological tissue remodelling hinders gas exchange and leads to permanent loss of lung functions (Wijsenbeek and Cottin. 2020). Histopathological findings performed on lung tissue samples obtained from patients with early phase COVID-19 pneumonia revealed similar fibroblastic foci as in usual interstitial pneumonia (Bhattacharya. 2022). According to transcriptomic analyses, COVID-19 associated lung injury share profibrotic CD163+ macrophages and alveolar type II cell cytopathic features with IPF (Sinha et al. 2022). Assuming that IPF and COVID-19 related ILD share several links to biological mechanisms and clinical behaviour, it was a logical step to consider anti-fibrotic treatment in a newly emerging diagnosis. Therefore, the primary intention and rationale for the use of antifibrotics is to avoid lung injury during the acute phase and subsequently to mitigate or prevent pulmonary functional impairment in a subgroup of patients with ongoing decline due to the development of progressive fibrosis. In addition to antifibrotics, several other compounds are in research and development as potential therapeutic agents in Post-COVID-19 pulmonary fibrosis (Table 3). Given the pathomechanism of COVID-19-associated ILD, innovative antifibrotics should meet that basic condition—to be focused preferably on the prevention of fibrotic remodelling in the early phase of lung damage due to SARS-CoV-2 infection more than on the mitigation of definitive changes.

Nintedanib is an orally active small molecule tyrosine kinase inhibitor that targets the platelet-derived growth factor (PDGF) receptor, vascular endothelial growth factor (VEGF) receptors and fibroblast growth factor (FGF) receptors (Wollin et al. 2015). Pirfenidone is an oral pleiotropic antifibrotic agent with multiple proposed pharmacodynamic mechanisms, comprising the regulation of pro-fibrotic and pro-inflammatory cytokine cascades and the inhibition of fibroblast proliferation, differentiation and synthesis of collagen. The central role rests in downregulation of TGF-β signalling (Oku et al., 2008; Tzouvelekis and Wolters. 2018).

Both small molecule drugs, pirfenidone and nintedanib, have demonstrated their ability to suppress several pro-fibrotic and pro-inflammatory pathways, resulting in the shift to fibrotic lung tissue conversion (Lederer and Martinez. 2018; Behr et al., 2021). Antifibrotics have been shown to slow progression in both IPF and non-IPF fibrotic ILD with progressive fibrosis behaviour. Accordingly, nintedanib has recently been approved for the treatment of other progressive fibrotic ILDs, whereas the efficacy and safety of pirfenidone has also been investigated in non-IPF progressive pulmonary fibrosis (Maher et al., 2020; Behr et al., 2021).

Several clinical trials currently listed on ClinicalTrials.gov are trying to reveal the potential role of nintedanib and pirfenidone in the management of pulmonary fibrosis following COVID-19 (Cherrez-Ojeda et al. 2022) either alone or in combination. In both original indications (IPF and progressive pulmonary fibrosis), clinical trials have confirmed that antifibrotic drugs need to be used for at least 1–3 months of treatment to have proven effect on lung function stabilisation or improvement (Molina-Molina et al., 2022; Richeldi et al., 2018). This fact must also be taken into account when considering any treatment schedule with nintedanib or pirfenidone.

Several published case reports have demonstrated the ability of nintedanib to accelerate lung recovery in critical COVID-19 with the need of invasive mechanical ventilation or extra-corporeal membrane-oxygenation (Bussolari et al., 2021; Ogata et al., 2021). In contrast, *Umemura et al.* carried out an interventional prospective study using a historical control group. Thirty patients were enrolled and treated with 150 mg of nintedanib administered twice daily from day 1 to liberation from mechanical ventilation over a period of 28 days. The authors concluded that the addition of nintedanib in patients already undergoing mechanical ventilation may not be beneficial in terms of the 28-day mortality rate despite improvements in the length of mechanical ventilation, high-attenuation areas on CT or oxygenation parameters (Umemura et al. 2021). Current antifibrotics, along with corticosteroids, might play a pivotal role in preventing or retarding fibrosis at an earlier stage of severe COVID-19 pneumonia (Kumar et al. 2021). However, with respect to the original indication, attention is focused primarily on nintedanib for the treatment of lung fibrosis in survivor cohorts after recovering from acute COVID-19. At least three clinical trials recently enrolled adult symptomatic patients suffering from fibrotic lung abnormalities and functional impairment. Various study endpoints include signs of slowing down lung fibrosis, HRQL/radiological/functional improvements under treatment, etc. (Bazdyrev et al., 2021; Cherrez-Ojeda et al., 2022).

Pirfenidone is a standard-of-care anti-fibrotic drug for fibrotic conditions such as IPF. In patients with IPF, pirfenidone at a daily dose of 2403 mg significantly reduced the rate of decline in forced vital capacity (FVC) (Lederer and Martinez. 2018; Noble et al., 2011; King et al., 2014) and improved survival. Similarly, pirfenidone also confirmed its ability to slow down the loss of lung functions in progressive unclassifiable fibrosing ILDs (Ghazipura et al. 2020). Ferrara et al. concluded that pirfenidone may reduce and prevent the subsequent immune-inflammatory response that precedes pulmonary fibrosis (Ferrara et al. 2020). Furthermore, the tailored timing of pirfenidone administration reflects the efficacy in the prevention of pulmonary fibrosis; on the other hand, due to its antifibrotic properties, pirfenidone would cure persistent pulmonary fibrotic changes (Seifirad. 2020; Kayarat et al., 2021). Not all expectations were fulfilled until now. In a recent trial from China, pirfenidone was unable to significantly improve the lung interstitial lesions in severe COVID-19 (according to CT analysis). However, some benefits in anti-inflammatory responses and prevention of thrombotic complications were achieved through pirfenidone treatment (Zhang et al. 2022). Several ongoing clinical trials intend to evaluate the effect of pirfenidone in patients with pulmonary fibrosis who have suffered from severe pneumonia due to COVID-19. At the end of November 2022, three active clinical trials for COVID-19-related ARDS or fibrotic lung disease after COVID-19 pneumonia were listed on the ClinicalTrials.gov web page. In addition, multiple case studies on this topic have already been published. Based on the scientific rationale, pirfenidone still represents a promising antifibrotic strategy for use in patients with acute severe SARS-CoV-2 infection, as well as during the post-acute period with imminent fibrotic pulmonary sequelae (Al-kuraishy et al. 2022).

## Discussion

Clinical evidence during the COVID-19 pandemic has shown that SARS-CoV-2 infection poses a threat for a non-negligible part of the population, considering the risk of severe respiratory disease on the background of immunological dysregulation (Amin et al., 2022; Stewart et al., 2023). Even if usually resolved, some cases of severe lung tissue injury may undergo an abnormal healing process and result in pulmonary fibrosis (van Gassel et al. 2021). As Post-COVID-19 pulmonary fibrosis represents a newly emerging entity, it is not surprising that long-term follow-up data are not yet available to the required extent and quality (Zheng et al. 2023). Therefore, increasing attention is being paid to research activities focused on various aspects of the disease—epidemiology, risk factors, pathogenesis, diagnosis, acute and long-term medical care. Anti-inflammatory, antiviral, antioxidant and antifibrotic properties of innovative and repurposed drugs may attenuate the increasing burden of Post-Covid-19 pulmonary fibrosis (Bazdyrev et al., 2021; Zheng et al., 2023)*.* Multiple clinical trials focused on pharmacological treatment regimens have been either designed or are already in progress. However, contrasting results have thus far been recorded due to various causes. It should be mentioned that we still lack a sufficient number and scope of randomised clinical trials, because those that have been published differ in design and do not always take into account the individual disease behaviour and diverse interventions. For obvious reasons, attention turns especially to antifibrotic drugs (e.g., nintedanib and pirfenidone) (Chaudhary et al., 2020; Bazdyrev et al., 2021; Mohammadi et al., 2022), although the absence of high-quality data continues to pose an unmet need for the future. The promising therapeutic value of nintedanib and pirfenidone in the COVID-19 scenario remains unclear for multiple reasons. Firstly, because there is currently no consensus regarding the selection criteria of treatment and spontaneous disease behaviour to resolve over time, reflecting inter-individual variability, cannot be ruled out. Secondly, dosing regimens and treatment duration have not been uniform across the reports published thus far. Moreover, the daily dose of antifibrotic drugs may have not always reached the recommended dose for original indication (IPF or non-IPF progressive pulmonary fibrosis). Thirdly, the co-administration of immunosuppressives or corticosteroids might affect treatment results (Bussolari et al., 2021; Ogata et al., 2021; Umemura et al., 2021).

Finally, we must take into account some other aspects that have emerged during the course of the pandemic: the impact of novel viral variants in the context of post-acute pulmonary sequelae, the influence of vaccination, as well as antivirals and, last but not least, antifibrotic treatment discussed above. Since the onset of the pandemic, numerous SARS-CoV-2 variants arose with changes in its genetic sequence. Different SARS-CoV-2 variants presumably influence the clinical outcomes in the affected population (Mendiola-Pastrana et al., 2022). For example, the expansion of the SARS-CoV-2 Omicron subvariants in populations with prevalent immunity gained from prior infection and/or vaccination has been associated with a weakened burden of severe COVID-19 pneumonia relative to the Delta variant (Lewnard et al. 2023). Vaccination led to a significantly milder severity of COVID-19 pneumonia based on CT scans in vaccinated compared to unvaccinated patients during both Delta and Omicron variant predominant periods. Similarly, booster or additional doses of vaccines were associated with differences even between vaccinated individuals (Wada et al. 2022). The recent cohort study found the antiviral nirmatrelvir/ritonavir to reduce the risk of Post-COVID-19 conditions regardless of vaccination status and prior infection history. This protective effect was also evident in pulmonary component (Xie et al. 2023). We suppose, that the risk modulation throughout the phases of the disease will translate into lower incidence of Post-COVID-19 pulmonary fibrosis in treated patients.

There is still a lack in understanding the long-lasting effects of severe viral respiratory infection in the context of COVID-19. The patients with COVID-19 pneumonia can fully recover or progress either to transient interstitial lung abnormalities or to Post-COVID-19 pulmonary fibrosis. To date, there are currently no long-term studies and no treatment guidelines to alleviate ILD associated with COVID-19. Lastly, many of the current, repurposed and emerging drugs could possess therapeutic benefits for treating and preventing the long-term fibrotic consequences of severe COVID-19.

## Future directions

The inter-individual diversity of origins together with similar pathophysiological mechanisms and qualitatively same clinical consequences creates space for research and development in the field of pharmacological treatment strategies directed at Post-COVID-19 pulmonary fibrosis. International multicentre collaborations with long term follow-up periods, in addition to ongoing institutional efforts, are undoubtedly required to address these unmet needs. Several strategies may be proposed to limit the risks related to the development and progression of COVID-19 associated ILD: prevention of infection (through vaccination), effective inhibition of viral replication (through antivirals), immunomodulation/restitution of normal immune response (through immunomodulators, anti-inflammatory or immunosuppressive agents) and the antifibrotic treatment (through multiple agents in development whose promising mechanism of action needs to be clarified in clinical setting).
